# Chagas Disease: Perspectives on the Past and Present and Challenges in Drug Discovery

**DOI:** 10.3390/molecules25225483

**Published:** 2020-11-23

**Authors:** Felipe Raposo Passos Mansoldo, Fabrizio Carta, Andrea Angeli, Veronica da Silva Cardoso, Claudiu T. Supuran, Alane Beatriz Vermelho

**Affiliations:** 1 BIOINOVAR-Biocatalysis, Bioproducts and Bioenergy, Institute of Microbiology Paulo de Góes, Federal University of Rio de Janeiro (UFRJ), Rio de Janeiro 21941-902, Brazil; mansoldo@micro.ufrj.br (F.R.P.M.); verocardoso@micro.ufrj.br (V.d.S.C.); 2 Neurofarba Department, Università degli Studi di Firenze, Sezione di Scienze Farmaceutiche, Via Ugo Schiff 6, 50019 Sesto Fiorentino (Florence), Italy; fabrizio.carta@unifi.it (F.C.); andrea.angeli@unifi.it (A.A.); 3 Centre of Advanced Research in Bionanoconjugates and Biopolymers Department, “Petru Poni” Institute of Macromolecular Chemistry, 700487 Iasi, Romania

**Keywords:** Chagas disease, *Trypanosoma cruzi*, proteochemometric, drug discovery, clinical trials

## Abstract

Chagas disease still has no effective treatment option for all of its phases despite being discovered more than 100 years ago. The development of commercial drugs has been stagnating since the 1960s, a fact that sheds light on the question of how drug discovery research has progressed and taken advantage of technological advances. Could it be that technological advances have not yet been sufficient to resolve this issue or is there a lack of protocol, validation and standardization of the data generated by different research teams? This work presents an overview of commercial drugs and those that have been evaluated in studies and clinical trials so far. A brief review is made of recent target-based and phenotypic studies based on the search for molecules with anti-*Trypanosoma cruzi* action. It also discusses how proteochemometric (PCM) modeling and microcrystal electron diffraction (MicroED) can help in the case of the lack of a 3D protein structure; more specifically, *Trypanosoma cruzi* carbonic anhydrase.

## 1. Introduction

Chagas disease (CD) or American trypanosomiasis is an infectious disease caused by the parasite *T. cruzi* transmitted by hematophagous triatomine bugs. The disease is endemic in Latin America but it is also found in non-endemic regions such as North America, Europe (Austria, Belgium, France, Germany, Italy, Netherlands, Portugal, Spain, Sweden, Switzerland and the United Kingdom), Japan and Australia due to international immigration, blood transfusion, organ transplantation, congenital infection and oral transmission through food [1]. According the Drugs for Neglected Diseases (DNDi), 6–7 million people are infected with the *T. cruzi* parasite; fewer than 10% of infected people are diagnosed, 33% of them present cardiac damage and 70 million people are at risk [2]. So far, a vaccine for CD is not available so primary prevention has been based on vector control and prevention of transmission through actions such as the compulsory screening of blood donors and the continuous application of insecticides in infested homes [3]. Despite advances in the control of domestic vector infestation since 1991, challenges still exist in more endemic areas and with extensive wild infestation such as in Gran Chaco and the Amazon Basin [4]. The chronic Chagas cardiomyopathy (CCC) is the most severe clinical manifestation of the disease that occurs years or decades after acute infection [5]. According to Vermelho et al., it is a disease considered neglected and few drugs are being developed for the treatment of CD with no progress in this direction since the 1960s [6]. Regarding the discovery of new drugs for CD, the strategy of target-based drug discovery was hampered by the lack of targets well validated [7]. According to Pérez-Molina and Molina, in the area of drug development, existing animal models are limited because they have a poor translation of in vivo data [8]. Therefore, according to the authors, it is necessary to standardize new animal models capable of more safely predicting the effectiveness of new drug candidates [8]. According to Thompson et al., the phenotypic screening of libraries with several compounds is still considered the best strategy to identify new leads or starting points [7]. Moreover, the discovery of new drugs for Chagas disease need collaborative networks involving academia, pharmaceutical companies, government organs and entities such as DNDi (Drugs for Neglected Diseases initiative), all of them contributing for substantial advances where actions must be as synergistic as possible in order to support the translation of academic research into available drugs [9,10]. Many of the methods used in the field of drug discovery are powerful and can offer important information about interactions and functions; however, they require access to the 3D structure of the protein in question [11]. According to Freyhult et al., the need for prior knowledge of the 3D structure is a bottleneck and therefore it is important to develop new methods that do not require the 3D model of the protein [11]. As a result, according to the authors, a new bioinformatic approach was created called proteochemometrics (PCM), whose strong point is the presentation of results obtained directly from data of real interactions and that does not require knowledge of the 3D protein structure model [11,12].

### 1.1. Current Drugs

#### 1.1.1. Benznidazole and Nifurtimox

**Figure 1:**
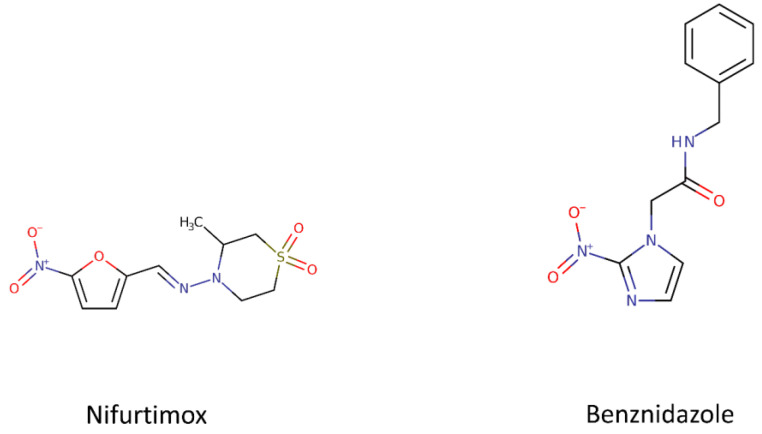
Chemical structures of the two nitroheterocyclic compounds developed for Chagas disease. The chemical structure of Nifurtimox and Benznidazole were retrieved from Drug Bank (http://www.drugbank.com).

Currently only two nitroheterocyclic compounds are available to treat Chagas disease and both of them were developed more than 50 years ago: Nifurtimox and Benznidazole (Figure 1). Besides the toxic effect and resistance, the treatment is long (60–90 days) and has not been effective in chronic patients. A new drug is needed that is safe and effective for both the acute and chronic stages of the disease but several factors are barriers to the development of new candidates. We can mention the lack of biomarkers for the two stages of the disease and for the evaluation of the therapeutic efficacy of the treatments as well as the genetic diversity of *T. cruzi* strains among others. It is also necessary to review the assays and tools used with in vitro and in vivo models for the translation studies [6,13].

In 1971, benznidazole (BNZ), N-benzyl-2-nitroimidazole acetamide, was released by Roche. Nowadays, Nortec Química (Brazil), LAFEPE (Brazil), Maprimed (Argentina) and Elea (Argentina), produce the BZN. Six years earlier, nifurtimox (NFX), 3-methyl-4-[5 9-nitrofurfurylideneamine] had been commercialized by Bayer under the trade name of Lampit. NFZ presents high toxicity and side effects such as hypersensitivity reactions, anorexia, vomiting, polyneuritis and depression. Due to these adverse reactions, this drug has become inconvenient for clinical use and is no longer used in most countries of Central and South America. BZN is the drug available and clinically prescribed for treatment although the occurrence of side effects such as hepatitis, peripheral polyneuropathy, digestive intolerance and anorexia leads to limitations on its use.

However, the benefits offset the risk and the treatment is still favorable especially in the acute phase. Benznidazole administration of 5–8 mg/kg/day for children or 5–10 mg/kg/day for adults during 30–60 days was the most frequent protocol of use [5]. The cure rates with BNZ is 60–100% in the acute phase and in younger people. In the chronic phase, the anti-parasitic effect is observed but the weak decrease in clinical symptoms shows limited cure effectiveness (8–20%) [14]. This was reinforced by the results of the BENEFIT trial, which showed that treatment with BNZ did not reduce clinical progression in patients with established advanced cardiomyopathy [15]. Although the mechanism of the action of BZN is not completely understood, BZN is activated by NADH-dependent trypanosomal reductases and forms reductive metabolites that, supposedly, cause a series of effects such as DNA damage and inhibition of protein synthesis [16]. In 2017, the FDA granted approval for the use of benznidazole in children aged between 2 and 12 years old with Chagas disease and was the first drug to be approved in the U.S. for the treatment of Chagas disease. Since the introduction of BZN and NFX, only allopurinol and triazoles (inhibitors of ergosterol biosynthesis) have been studied in clinical trials, observational studies and case reports [17].

#### 1.1.2. Drug Discovery for Chagas Disease: A Challenge

The drug discovery process for Chagas disease has been ongoing with thousands of compounds tested annually before finding a promising candidate. High throughput screening (HTS) campaigns, which represent an important approach to identify new sets of leads for treatment, have been carried out [10]. The DNDi is developing a portfolio of early hits and lead series with collections of natural products and synthetic compounds to find new drug candidates for Chagas disease. In 2015, the Neglected Tropical Diseases (NTD) Drug Discovery Booster was launched with several pharmaceutical companies and this project is ongoing [18]. In 2019, the DNDi launched the ‘Chagas Hit-to-lead’ project with the objective of identifying new leads with activity in animal models with the disease and also established a new consortium in collaboration with the University of Campinas and the University of São Paulo in Brazil [19]. Table 1 shows a summary of the drugs studied as a treatment for Chagas disease listed in the Clinical Trials database (https://clinicaltrials.gov/) and the results are organized by their respective status.

## 2. Drugs, Targets and Inhibitors

Several targets are being studied for the development of new drugs and a few of them will be cited in this review. In recent years, factors that have hindered the development of new drugs have been extensively discussed in the literature. The lack of accurate biomarkers for treatment, failure in diagnoses, diversity of strains of the parasite, problems with the standardization of methodologies such as in vivo animal models, different host cell culture lines and problems in the translation process among other factors are barriers for the development of new drugs [6,13,20,21].

### 2.1. Ergosterol Pathway and Inhibitors of CYP51

The *T. cruzi* ergosterol pathway has been extensively studied in the search for new drugs. Ergosterol is essential for the trypanosome membranes and is required for parasite multiplication. The main enzymes of this pathway have already been studied and sterol-14-alpha demethylase (CYP51) proved to be essential for the parasite’s viability. CYP51 was tested as a therapeutic target for CD and in this context anti-fungal triazole derivatives that inhibit this enzyme such as ravuconazole, prodrug E1224 (Fosravuconazole), VNI [(R)-N-(1-(2,4-dichlorophenyl)-2-(1H-imidazol-1-yl)ethyl)-4-(5-phenyl-1,3,4-oxadiazol-2-yl)benzamide)] [22], VFV [(R)-N-(1-(3,4′-difluoro-[1,1′-biphenyl]-4-yl)-2-(1H-imidazol-1-yl)ethyl)-4-(5-phenyl-1,3,4-oxadiazol-2-yl) (benzamide] [23] and posaconazole exhibited properties suitable for the treatment of CD. Posaconazole, ravuconazole and its prodrug E1224 belong to DNDi projects. Monotherapy with ravuconazole or posaconazole was not effective for the treatment of chronic CD [24]. E1224 proved to be effective in eliminating the parasite at the end of treatment but there was limited sustained efficacy one year after treatment as a single medicine. These results compared with BZN alone were worse in the Chagas parasite eradication rates [25]. Moraes et al. demonstrated that a few *T. cruzi* genotypes (DTUs) are partially resistant to four ergosterol biosynthesis inhibitors (posaconazole, ravuconazole, EPL-BS967 and EPL-BS1246) [26]. Villalta et al. reported the first experimental cure for Chagas disease by VNI [22]. Using murine models infected with the Y strain of *T. cruzi*, treatments with NIV and VFV resulted in 100% animal survival and 0% natural recurrence after the end of therapy. VFV was more potent in both sexes, causing a reduction in peak parasitemia > 99.7% (Benznidazole was the reference drug). Guedes-da-Silva et al. and Soeiro et al. reported a high anti-parasitic efficacy of VNI and its derivative (VNI/VNF) against both forms of *T. cruzi* that were relevant to the host infection mammals (bloodstream and amastigotes) with in vivo potency at 25 mg/kg twice a day (bid), similar to that of benznidazole (100 mg/kg/day) [27,28]. According to Villalta and Rachakonda, rigorous PK/PD analysis is essential to translate the results of preclinical in vivo studies and that both inhibitors (VNI and VFV) are potential promising candidates for clinical trials [17]. Until the present, the DNDi discouraged the use of new azoles that target the *T. cruzi* sterol 14 α-demethylase due to the failure of the clinical trial.

### 2.2. Carbonic α-Anhydrase and the Inhibitors Sulfonamides, Thiols and Hydroxamates

The carbonic α-anhydrase (TcCA) of *T. cruzi* is characterized as a target for action of new drugs. This enzyme is associated with growth factors and virulence factors for pathogens and are involved in mechanisms including breathing, CO_2_ and bicarbonate transport, pH regulation, electrolyte secretions and biosynthetic reactions [29,30,31,32]. In *T. cruzi*, an α-CA was identified, cloned and characterized. TcCA has a high catalytic activity for the CO_2_ hydration reaction [33,34]. Although the role of the enzyme in the pathogen is still poorly understood, the inhibition of TcCA is an important factor for eliminating the parasite, resulting in damage in the mechanisms of ion exchange and a strong reduction of the metacyclogenesis capacity by the parasite and the inhibition of growth. These biological events will lead the parasite to death due to the large amount of stress suffered [35]. In the last decade, several inhibitors of *T. cruzi* carbonic α-anhydrase were discovered; these compounds with structures containing sulfonamides, thiols and hydroxamates have a high capacity for binding with zinc and the inhibition of its activity [31,36,37,38]. This assumption was supported by a few in vitro studies that demonstrated the ability of thiols and hydroxamates to inhibit the three phases of the pathogen’s life cycle [31]. Based on these studies, TcCA is emerging as a new and promising therapeutic target [21]. Llanos et al. used a structure-based approach to identify new compounds that inhibit *T. cruzi* carbonic anhydrase (TcCA) where 10 compounds from 255 were selected for testing against TcCA [21]. The authors attested that the combination of computational methodologies allowed the finding of high potency compounds with KI values in the nanomolar range and were selective to inhibit TcCA (which have trypanocidal effects against *T. cruzi* epimastigotes and trypomastigotes). As a result, they reported the discovery of new TcCA inhibitors with a poor interaction with human carbonic anhydrase (hCA), of which two molecules are commercial artificial sweeteners that have vast toxicological data available. Finally, the authors reported that further investigations are needed for a deeper understanding of selectivity against TcCA.

In this regard, with respect to the interaction between the inhibitor and the enzyme, Clabbers et al. developed a method to visualize the binding interaction of a sulfonamide inhibitor to human carbonic anhydrase isoform II (hCA II) [39]. In this work, the authors used microcrystal electron diffraction (MicroED), which is a method capable of determining the structure of proteins, peptides and small organic molecules, in many cases at very high resolutions [40]. MicroED is a new frontier after X-ray crystallography and is capable of being used in small proteins such as CAs and inhibitors. Thus, MicroED can become a new tool in drug discovery experiments, complementing structural biology methods such as x-rays and neutron diffraction [39]. As a result, the authors found that the data generated were of high quality and served to adjust and resolve the inhibitor bound to the active site of hCA II.

**Figure 2:**
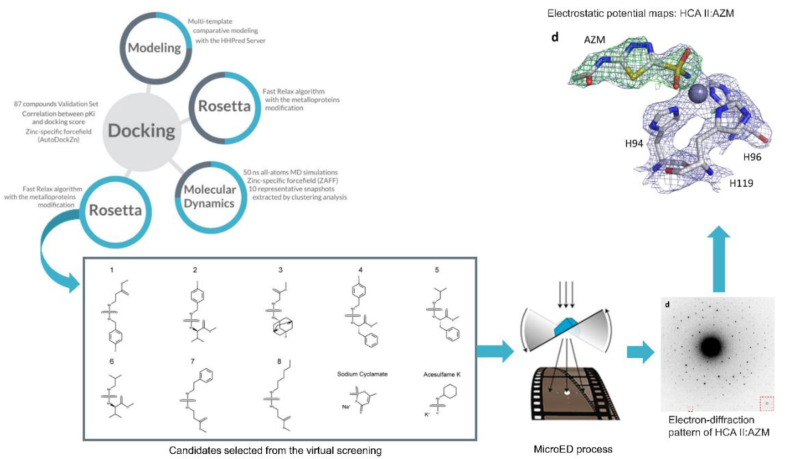
Scheme proposed for coupling the workflow used by Llanos et al. [21] with the MicroED technique applied by Clabbers et al. [39]. For illustrative purposes, the human carbonic anhydrase (hCA) image was used; however, the idea of the scheme is this application for studying TcCA with the inhibitors selected by the workflow proposed by Llanos et al. [21]. The MicroED process illustration is from the work of Nannenga and Gonen [41].

Therefore, the use of MicroED for the analysis of carbonic anhydrase inhibitors appears to be promising. Due to the successful use of MicroED in hCA II, a new scheme can be proposed as shown in Figure 2 where MicroED is coupled to the workflow proposed by Llanos et al. [21]. For illustrative purposes, the hCA II image of the authors’ work was used; however, the proposal is that future works apply this coupling strategy with TcCA. In this way, the proposed workflow of comparative modeling, molecular dynamics and docking simulations can take advantage of the high resolution of the results obtained by MicroED.

Based on this evidence, it is worth highlighting the work authored by D’Ambrosio et al. [42]. According to the authors, the most promising results for TcCA inhibitors are thiol and hydroxamate compounds and they have also evaluated sulfonamides, metal-complexing anions, halogenides and pseudohalogenides [42]. However, this field is still in its infancy, requiring additional studies to understand the role of CAs in the *T. cruzi* cycle as well as the genetic validation of this target [42]. According to Pereira et al., a drug discovery strategy considers genetic validation as a critical point; however, *T. cruzi* does not have RNA interference and the traditional genetic knockouts are often not successful [43]. Another problem is that these genetic experiments are often carried out in the insect phase of the parasite. Therefore, a biological effect is not often observed in the other stages of the insect’s development [43]. Finally, the authors suggest that CRISPR-Cas9 may be a promising tool for the genetic validation of trypanosomatid targets, contributing significantly to the area.

According to a review by Supuran, several works have studied in detail the inhibition of the enzyme α-carbonic anhydrases (CAs, EC 4.2.1.1) of *T. cruzi* using anion compounds, sulfonamides, sulfamates, thiols and hydroxamates. Due to the success of some results, TcCA inhibition represents a new mechanism of action, being a target with few studies on obtaining anti-Chagas disease agents [31]. More recently, according to Supuran, several studies have reported success in inhibiting TcCAs in vitro and a few have also shown the inhibition of parasite’s growth. However, as TcCA has not been crystallized yet, the main challenge is the selectivity for inhibiting pathogenic over host enzymes [44].

### 2.3. Tc80 Proteinase and Peptides

*T. cruzi*, in the infective trypomastigote and replicative intracellular amastigote form, secretes the enzyme prolyl oligopeptidase 80 kDa (Tc80), which apparently is related to the invasion of non-phagocytic cells [45,46]. Tc80 proteinase is responsible for degrading extracellular collagens of the infected cell matrix and is one promising target. The peptides Peptidyl nitrile and Peptidyl ketobenzothiazole are reversible and competitive inhibitors of Tc80 [47,48].

### 2.4. Cysteine Peptidase and K777

Cruzipain is a lysosomal cysteine peptidase expressed in all evolutive forms of the parasite. K777 is a vinyl sulfone derivative inhibitor; it is considered the most potent and well-known inhibitor for this enzyme. In vivo, K777 did not promote a parasitological cure but significantly reduced parasite-induced heart damage. A wide range of susceptible and resistant strains was sensible to this inhibitor. Although K777 entered in preclinical development, the study was stopped. An HTS study conducted by GlaxoSmithKline identified new inhibitors against cysteine peptidase and these studies are in progress [49]. Therefore, this class of inhibitors is being considered a promising drug for CD.

### 2.5. Proteasome Inhibitors

The proteasome was identified as a promising drug target for kinetoplast diseases such as leishmaniasis, Chagas Disease and African trypanosomiasis (HAT). It is considered a key component of the ubiquitin-proteasome protein degradation system and plays an important role in many cellular processes. GNF6702 was invented by researchers working at the Genomics Institute of The Novartis Research Foundation in 2013, with activity against these diseases. Another inhibitor, the GSK3494245/DDD01305143, is a preclinical candidate for visceral leishmaniasis developed from a *T. cruzi* screening hit [50,51,52,53]. Recently, new *T. cruzi* proteasome inhibitors using a luminescence-based high throughput screening assay have been identified [52].

## 3. Computational Methods

The process of developing a drug can take about 12 years or more from development to approval for launching the drug on the market [54]. According to Shen et al., the traditional drug discovery process is largely based on high throughput screening (HTS), which is an acceptable performance technique but of high cost and low efficiency [55]. As the traditional drug discovery process has a high cost and high failure rate, there was a need to use techniques based on computer-aided drug discovery (CADD), which included ligand-based (e.g., Cheminformatics), structure-based (e.g., Molecular docking) and systems-based (e.g., Proteochemometric modeling) drug design [56]. According to Schaduangrat et al., the role of CADD is to select a library of compounds in relation to a target of interest, starting with the identification of target or hit compound using results from wet-lab experiments and, later, via HTS [56].

Structure-based drug design (SBDD) is an approach [57] that depends on knowledge of the 3D structure of the biological target in question, being a computational approach that assists in the main phases of drug discovery such as hit identification and lead identification [58]. According to Kalyaanamoorthy and Chen, these two phases comprise the identification of a series of chemical compounds (hits) that ideally have some degree of action and specificity against the target and subsequently evaluated the selection that was carried out to identify promising molecules (leads) [58]. In SBDD, two methods are frequently used, namely, molecular docking and de novo ligand design [59]. Molecular dynamics (MD) is a technique used to study the dynamic behavior of macromolecules [60] and is often used in SBDD to provide information about protein dynamics, how ligands bind to target proteins, interaction pathways, unravel novel cryptic binding sites and conformational change events [60,61].

The discovery of an inhibitor to a target that drives the phenotype of a disease is one of the most important phases of a drug discovery campaign [62]. As this is an expensive and slow process, computational methods are increasingly being sought to accelerate this optimization phase for predicting protein-ligand binding affinity values [62]. Screening of compound libraries is expensive and time-consuming so the Quantitative structure–activity relation (QSAR) method is an alternative for selecting lead molecules [63]. However, conventional QSARs take into account the interactions of multiple compounds with only a single target, thus having some disadvantages such as predictive power limited by the amount of data on a specific target and difficulty in identifying new classes of ligands or new patterns of the binding of similar compounds that are outside the training set [64]. In order to circumvent QSAR deficiencies, a new approach was invented by Lapinsh et al. and is entitled proteochemometric (PCM) modelling [65], being an extension of the QSAR [64]. According to van Westen et al., unlike QSAR, PCM is a modeling that is based on the similarity of a group of ligands and a group of targets, modeling the space of interaction between the ligand-target as it takes into account the chemical descriptors of the compounds added to the descriptor of the protein or target [66]. Another advantage of PCM according to Schaduangrat et al. is that this method does not require knowledge of the three-dimensional structure of the protein, requiring only the sequence of amino acids [12]. The term ‘proteochemometric’ is most often used in studies that analyze the impact of protein and molecule descriptors on prediction performance [67]. On the other hand, the term ‘chemogenomics’ is preferable in studies to predict the specificity of drugs on a large scale in the protein space [67].

The TDR Targets Database (http://tdrtargets.org) is an open access database focused on identifying and prioritizing molecular targets for the development of drugs for neglected human diseases [68]. The system used on the website uses the chemogenomics approach that links target genes to suitable chemical inhibitors in addition to making other relationships available on the website, which currently has 5300 druggable targets, 2,000,000 bioactive compounds, 7,200,000 bioactivities, 45 full proteomes, 20 genome-wide prioritizations and 1,200,000 annotations [69]. In a study by Valera-Vera et al., the authors used a combined virtual screening strategy in the search for *T. cruzi* enolase inhibitors [70]. The search for a potential drug target was performed through a screening in the TDR Targets Database (v5) and the combined search strategy was based on ligand-based virtual screening performed on the Sweetlead database and target-based virtual screening using the ZINC database. As a result, the authors demonstrated that enolase can be a promising target for the treatment of Chagas disease and that etidronate can be a candidate as a drug for this treatment.

One possible strategy to accelerate drug discoveries is to analyze a new use for existing and already approved drugs. This strategy is known as drug repositioning or drug repurposing [71]. Alberca et al. used a cascade ligand- and structure-based virtual screening approach to identify compounds with a trypanocidal effect through the inhibition of putrescine uptake [72]. With a focus on drug repositioning, the authors used the DrugBank and Sweetlead databases for screening and thus reported for the first time the trypanocidal effects of butoconazole (and anti-fungal) and cinnarizine and meclizine. In a recent review by Bellera et al. on repositioning drugs for Chagas Disease using computer-aided technologies, the authors reported that these applications are still scarce and have only started to be used in the last five years [73]. Therefore, after examining several studies in this area, the authors concluded that the drug repositioning strategy has not yet been fully explored.

The development of drugs for neglected tropical diseases has been hampered due to the lack of well-characterized and validated targets [74]. According to Chatelain and Ioset, there are two approaches to drug development that are usually carried out to identify compounds of interest, namely, a target-based approach and a phenotypic-based approach, both with their advantages and limitations [74]. Among the two, phenotypic screening assays show impartial and more relevant results. On the other hand, results from a target-based approach are more rational when there is a robust target, making it easy to improve new compounds based on 3D docking studies [75]. According to Martínez-Peinado et al., among the possible existing strategies to identify compounds with activity against *T. cruzi*, the whole-cell phenotypic assay is generally preferred due to its higher translational rate to in vivo efficacy assessment when compared with a target-based approach [10]. Whole cell parasite screening is feasible in a high throughput screening mode and has several advantages such as the screening of large libraries the selection of compounds that show activity against the entire cell and selectivity analysis using mammalian cells, allowing the filtering of compounds that show general cytotoxicity [76]. Aulner et al. conducted a review on next generation phenotypic screening where the authors demonstrated that new methods based on transcript quantification, public databases and machine learning are collaborating to increase the results of phenotypic screening [77]. Thus, according to the authors, the phenotypic screening strategy begins to be considered as a catalyst in the discovery of drugs for infectious diseases. In a study by Ekins et al., the authors demonstrated that the combination of chemoinformatics and bioinformatics for *T. cruzi* drug discovery may result in the discovery of molecules with in vivo activity that previously might not have been selected [78]. For this, the authors used data from several public databases as well as compiled and curated biological and chemical compound screening data. As a result, it was found that the anti-malarial pyronaridine was effective in the acute Chagas mouse model and provided a new starting point for future research and optimization.

Based on the power of phenotypic screening in the HTS system, it is possible to carry out assays analyzing a large number of molecules for drug discovery. In this sense, Roquero et al. performed a high throughput phenotypic screen of a 150,000-compound library against *T. cruzi* and *Leishmania donovani* [79]. The authors opted for an open access disclosure of the screening campaign where they identified and characterized 12 new chemical series, seven of which were active against *T. cruzi* and *L. donovani*. According to the authors, the dissemination of hit structures and the associated activity can contribute to the drug discovery community [79]. Data sharing, open databases and computational tools have already proved useful in the study of drugs for Chagas disease as can be seen in the works of the Broad Institute and Collaborative Drug Discovery Inc. [77]. However, as highlighted by Aulner et al., although phenotypic high throughput screens are powerful, it is necessary to develop new tools and methods that support the management, data annotation, validation and sharing of the generated data so that the parasitology community can contribute and benefit from these advances [77].

## 4. Conclusions

Despite the efforts of several research groups and institutions, Chagas disease remains without an effective solution. The review of the latest drug discovery studies has shown that there are promising new targets as well as new drug candidates. However, ongoing and completed clinical trials have shown that there are few innovative options, where most trials use drugs that are already known for the treatment of Chagas disease. The development of computational tools with new algorithms that rely on increasingly powerful computers has shown promise in supporting drug discoveries. Limitations such as the lack of knowledge of the 3D structure of target proteins can be overcome with proteochemometric (PCM) modeling and microcrystal electron diffraction (MicroED), opening up new possibilities in the discovery of molecules of interest. Studies that used target-based and phenotypic-based approaches are increasingly based on computational methods; however, it has been demonstrated that technological advancement must be accompanied by an integration between systems, annotations and data availability so that the community that studies Chagas disease can, in an integrated way, contribute and benefit.

## Figures and Tables

**Table 1:** Summary of the main clinical trials with studies on drugs for the treatment of Chagas disease.

NCT Number	Acronym	Status	Interventions	Phases	Estimated Enrollment	Funded By	Locations
NCT02625974	CHICO	Active, not recruiting	Nifurtimox (Lampit, BAYA2502), Placebo	Phase 3	330 participants	Industry	Argentina, Bolivia, Colombia
NCT03334838		Completed	Nifurtimox (Lampit, BAYA2502)	Phase 1	36 participants	Industry	Argentina
NCT03350295		Completed	Nifurtimox (Lampit, BAYA2502)	Phase 1	48 participants	Industry	Argentina
NCT02606864		Completed	Nifurtimox (BAYa2502)	Phase 1	36 participants	Industry	Argentina
NCT01927224		Completed	Nifurtimox (BAYa2502)	Phase 1	37 participants	Industry	Argentina
NCT03892213		Completed	Benznidazole, E1224	Phase 1	28 participants	Other	Argentina
NCT03587766	FEXI12	Completed	Fexinidazole, Placebo Oral Tablet	Phase 2	45 participants	Other	Spain
NCT01377480	STOP CHAGAS	Completed	Posaconazole, Placebo for posaconazole, Benznidazole	Phase 2	120 participants	Industry	Argentina, Chile, Colombia, Guatemala, Mexico, Spain
NCT01162967	CHAGASAZOL	Completed	Benznidazole, Posaconazole	Phase 2	78 participants	Other	Spain
NCT02154269		Completed	Treatment with G-CSF (Granulocyte colony stimulating factor), Placebo saline	Phase 2	70 participants	Other	Brazil
NCT02386358	TRAENA	Completed	Benznidazole, Placebo	Phase 3	910 participants	Other	Argentina
NCT00123916	BENEFIT	Completed	Benznidazole, Placebo	Phase 3	2854 participants	Other	Argentina, Bolivia, Brazil, Colombia, El Salvador
NCT00323973		Completed	Bisoprolol	Phase 3	500 participants	Other	Colombia
NCT01755403	CINEBENZ	Completed	Benznidazole	Phase 4	52 participants	Other	Spain
NCT01549236	Pop PK Chagas	Completed	Benznidazole 12,5mg or 100mg	Phase 4	80 participants	Other	Argentina
NCT01557140		Completed	RASi plus carvedilol	Phase 4	42 participants	Other	Brazil
NCT03981523	TESEO	Recruiting	Benznidazole, Nifurtimox	Phase 2	450 participants	Other, NIH	Bolivia
NCT03704181	COACH	Recruiting	Colchicine 0.5 MG twice day for one year, Placebo Oral Tablet	Phase 2	60 participants	Other	Brazil
NCT04024163		Recruiting	Benznidazole	Phase 3	164 participants	Industry, Other	Argentina, Bolivia, Colombia
NCT00875173	STCC	Recruiting	Selenium, Placebo (for Selenium)	Phase 3	130 participants	Other	Brazil
NCT03672487	BETTY	Recruiting	Benznidazole, Placebo Oral Tablet	Phase 3	600 participants	Other	United States, Argentina
NCT03193749	ATTACH	Recruiting	Amiodarone Hydrochloride, Placebo Oral Tablet	Phase 3	200 participants	Other	Colombia
NCT01650792	CLINICS	Recruiting	Aspirin	Phase 4	500 participants	Other, NIH	Brazil
NCT04023227	PARACHUTE-HF	Recruiting	Sacubitril/valsartan, Enalapril	Phase 4	900 participants	Industry	Argentina, Brazil
NCT01489228		Unknown status	E1224, Benznidazole, Placebo	Phase 2	230 participants	Other/Industry	Bolivia
NCT03191162	MULTIBENZ	Unknown status	Benznidazole	Phase 2	240 participants	Other	Argentina, Brazil, Colombia, Spain
NCT03378661	BENDITA	Unknown status	Benznidazole, E1224, E1224 Placebo, Benznidazole Placebo	Phase 2	210 participants	Other	Bolivia
NCT02498782		Unknown status	Fexinidazole, Placebo	Phase 2	140 participants	Other	Bolivia
NCT02369978	CHICAMOCHA-3	Unknown status	Nifurtimox, Benznidazole, Placebo	Phase 2, Phase 3	500 participants	Other	Colombia
